# Diversity of *Blastocystis* Subtypes in Horses in Colombia and Identification of Two New Subtypes

**DOI:** 10.3390/microorganisms10091693

**Published:** 2022-08-24

**Authors:** Sohyeong Baek, Jenny G. Maloney, Aleksey Molokin, Nadja S. George, Jesús A. Cortés Vecino, Monica Santin

**Affiliations:** 1 Environmental Microbial and Food Safety Laboratory, Agricultural Research Service, United States Department of Agriculture, Beltsville, MD 20705, USA; 2 Laboratorio de Parasitología Veterinaria, Departamento de Salud Animal, Facultad de Medicina Veterinaria y de Zootecnia, Universidad Nacional de Colombia-Sede Bogotá, Bogotá D.C. 111321, Colombia

**Keywords:** *Blastocystis*, Colombia, epidemiology, horses, Illumina, MinION, NGS, subtypes

## Abstract

*Blastocystis* is a common intestinal protist in humans and animals worldwide. Wild and domestic animals are thought to be reservoirs of *Blastocystis* subtypes that also infect humans. There are limited studies on the prevalence and subtype distribution of *Blastocystis* in horses. In this study, 185 fecal samples were collected from horses (1 month to 17 years of age) in four regions of Colombia (Sabana de Bogotá, Costa Atlántica, Llanos Orientales, and Bogotá D.C.). *Blastocystis* presence and subtypes were determined by PCR and next generation amplicon sequencing. Eighty-one (43.8%) horses were positive for *Blastocystis*, with positive horses in all four regions. Molecular characterization identified 12 *Blastocystis* subtypes, 10 known subtypes (ST1, ST3–ST6, ST10, ST14, ST25, ST26), and 2 novel subtypes (ST33 and ST34). The validity of the novel subtypes was confirmed via phylogenetic and pairwise distance analyses of the full-length *SSU* rRNA gene sequences. Mixed subtype infections were common (55.6% of *Blastocystis*-positive horses). ST10 was the most prevalent subtype, present in 82.8% of *Blastocystis*-positive horses. Potentially zoonotic subtypes were identified in 88.9% of the *Blastocystis*-positive horses. This constitutes the most comprehensive study of *Blastocystis* in horses. Our findings indicate that horses harbor potentially zoonotic subtypes and could contribute to the transmission of *Blastocystis* to humans.

## 1. Introduction

*Blastocystis* is one of the most common intestinal eukaryotes found in humans [1]. Infections have been reported as associated with gastrointestinal symptoms and/or urticaria as well as asymptomatic [2,3,4]. *Blastocystis* has also been detected in a wide range of animals, suggesting its zoonotic potential and that animals may be a source of human infections [5]. A wide genetic diversity has also been identified within *Blastocystis* specimens isolated from mammals and birds, which has been described based on polymorphisms in the small subunit ribosomal RNA (*SSU* rRNA) gene. To date, 28 genetic variants, called subtypes (STs), meet the recommended conditions for unique subtype designation (ST1–ST17, ST21, and ST23–ST32) [6,7,8,9]. Twelve subtypes (ST1–ST10, ST12, and ST14) have been reported in both humans and animals, indicating the possibility of zoonotic and reverse zoonotic transmission [5,10,11,12]. *Blastocystis* transmission occurs via the fecal–oral route, with consumption of cysts either via direct contact with infected hosts or indirectly by ingestion of contaminated food or water [5].

Equines play an important role within human culture and are used for working purposes in industry, agriculture, and transport and for leisure or sporting activities. The Food and Agriculture Organization of the United Nations (FAOSTAT) estimates that the number of horses in the world is around 60 million [13]. Horses can carry zoonotic pathogens such as *Cryptosporidium* or *Giardia* [14,15,16,17]. Data on *Blastocystis* in horses is scarce. To date, there are eight published studies that tested for *Blastocystis* in horses and most examined few samples [7,18,19,20,21,22,23]. Only two of these studies detected *Blastocystis* in horses [7,18]. Of eight horses tested for *Blastocystis* at an army base in Thailand, one horse was positive [18]. A second study identified *Blastocystis* in 6 of the 11 horses examined in Colombia [7]. Better knowledge of subtypes of *Blastocystis* in horses is necessary to understand their potential role in *Blastocystis* transmission dynamics and as reservoirs for human infections. Therefore, the current study aimed to investigate the presence of *Blastocystis* subtypes in horses using next generation amplicon sequencing (NGS). Additionally, long-read sequencing using Oxford Nanopore Technology’s MinION was used to obtain the full-length *SSU* rRNA gene of two novel *Blastocystis* sequences identified in horses in this study, to confirm their validity as novel subtypes.

## 2. Materials and Methods

### 2.1. Source and Collection of Specimens

For this study, 185 fecal samples were collected from horses from four geographic regions in Colombia, South America (Bogotá D.C, Sabana de Bogotá, Llanos Orientales, and Costa Atlántica) from August to October 2007 (Table 1). The horses ranged from 1 month to 17 years of age, included males and females, and generally appeared in good health. Horses from the Sabana de Bogotá were “Silla Argentina” breed. Males were housed in separate stables, and their drinking water was from the nearest municipal aqueduct. Females were pastured with colts, with direct access to natural spring water. Horses from Costa Atlántica were “Paso Fino Colombiano” breed. Males were housed in separate stables, while females were pastured with colts. Both males and females consumed natural spring water. Horses from Bogotá D.C. that belong to the Universidad Nacional de Colombia were used for academic practices and had access to water from the Bogotá D.C. aqueduct. Horses from Los Llanos Orientales were almost wild, with direct access to natural spring water; they are only grouped together once or twice a year for examination and care. Feces were collected directly from the rectum of each horse and were placed into a 50 mL centrifuge tube with records of the date, location, age, gender, and identification and were transferred to an insulated container packed with ice or cold packs. Fecal specimens were shipped overnight to the USDA, ARS (Beltsville, MD, USA).

### 2.2. Parasite Concentration from Feces and DNA Extraction

To concentrate parasites, each fecal specimen was sieved and concentrated using a cesium chloride gradient, as previously described [24]. Genomic DNA was extracted using the DNeasy Tissue Kit (Qiagen, Valencia, CA, USA), in accordance with the recommendations of the manufacturer, with minor exceptions. Modifications included incubation with proteinase K (20 mg/mL) at 55 °C overnight and elution in 100 μL of AE buffer to increase the quantity of recovered DNA.

### 2.3. Molecular Detection and Subtype Identification Using Next Generation Amplicon Sequencing

A next generation amplicon sequencing strategy was used to detect *Blastocystis*, as previously described [25]. Briefly, a PCR using primers ILMN_Blast505_532F and ILMN_Blast998_1017R was used to screen all 185 horse samples. These primers amplify a fragment of the *SSU* rRNA gene (*ca.* 500 bp) and are identical to Blast505_532F/Blast998_1017R [26], with the exception of containing Illumina overhang adapter sequences on the 5′ end. Final libraries were quantified by Qubit fluorometric quantitation (Invitrogen, Carlsbad, CA, USA) prior to normalization. A final pooled library concentration of 8 pM with 20% PhiX control was sequenced using an Illumina MiSeq and a 600 cycle v3 kit (Illumina, San Diego, CA, USA). Paired end reads were processed and analyzed with an in-house pipeline that uses the BBTools package v38.82 [27], VSEARCH v2.15.1 [28], and BLAST+ 2.11.0 [29]. Briefly, read pairs were merged, filtered for quality and length, denoised, and checked for chimeric sequences. Clustering and the assignment of centroid sequences to operational taxonomic units (OTU) was performed within each sample at a 98% identity threshold. Only those OTUs with a minimum of 100 sequences were retained and then checked for chimeras once more. OTUs were then blasted against *Blastocystis* references from NCBI. All hits below an alignment length of 400 bp were removed. Raw FASTQ files were submitted to NCBI’s sequence read archive under project PRJNA856306 and accession numbers SRR20005885–SRR20005965. The nucleotide sequences generated using NGS in this study were deposited in GenBank under the accession numbers ON932503–ON932559.

### 2.4. PCR Amplification and Sequencing of the Full-Length SSU rRNA Gene

DNA from three horse samples (d31, d43, and d45) were used to obtain full-length SSU rRNA gene sequence to validate novel subtypes ST33 and ST34. A previously described PCR and Nanopore sequencing strategy was used to generate sequences of the approximately 1800 base pair *SSU* rRNA gene [30], with the following updates. Briefly, a PCR using the MinION-tailed primers SSU-F1 (5′–TTT CTG TTG GTG CTG ATA TTG C AAC CTG GTT GAT CCT GCC AGT AGT C−3′) and SSU-R1 (5′–ACT TGC CTG TCG CTC TAT CTT C TGA TCC TTC TGC AGG TTC ACC TAC G−3′), which amplify most eukaryotic organisms’ full-length *SSU* rRNA gene, was performed using the high-fidelity proofreading polymerase contained in KAPA HiFi HotStart ReadyMix (KAPABioSystems, Cape Town, South Africa). Initial denaturation was performed at 98 °C for 5 min followed by 35 cycles of amplification: 20 s at 98 °C, 45 s at 60 °C, and 90 s at 72 °C. The final extension continued for 5 min. PCR amplicons were purified using a 0.5X AMPure XP beads (Beckman Coulter, Brea, CA, USA) to sample ratio and quantified on a Qubit Fluorometer (ThermoFisher Scientific, Waltham, MA, USA). To prepare the nanopore sequencing library, the Oxford Nanopore Technologies (ONT) SQK-LSK110 Ligation Sequencing Kit was used in accordance with the protocol of the manufacturer for PCR Barcoding Amplicons (PBAC12_9112_v110_revB_10Nov2020) and loading guidelines for R10.3 flow cells. The EXP-PBC001 PCR Barcoding Kit (ONT, Oxford, UK) was used in combination with the ligation kit for barcoding each sample. A modification to the barcoding PCR protocol included the use of the KAPA HiFi polymerase, as described above, instead of the NEB LongAmp Taq. Amplicons were purified with 0.5X XP beads once more, quantified, and diluted to ensure 100 fmol of library in 12 µL was loaded onto a flow cell and run aboard an Mk1C MinION using MinKNOW v21.11.7 software.

Basecalling was performed using Guppy v6.1.1 (gpu) and the Super Accuracy model available for R10 flow cells in the following configuration files dna_r10.3_450bps_sup.cfg. A minimum quality score cutoff of 10 was used for filtering reads. FASTQ reads were then length-filtered to include only reads between 1600 and 2000 nucleotides. Filtered reads were corrected using canu v2.2 and then length-filtered again to retain reads between 1600 and 2000 nt. Next, MinION PCR adapters were trimmed and only reads with intact forward and reverse eukaryotic primers were retained (bbduk.sh k = 18 restrictleft/right = 150 mm = f edist = 2; BBTools v38.94). Primer orientation was used to ensure all reads were converted to the plus strand, before combining them into a single FASTA file. Reads were then clustered using the vsearch –cluster_fast command (vsearch v2.15.1) with a 98% identity threshold and checked for chimeras using the vsearch –uchime_denovo command. The chimera-free clusters were then polished using Racon v1.4.20. The SAM file needed for racon polishing was generated by first extracting non-chimeric reads from pre-clustered reads and mapping them back to the chimera-free clusters using Minimap2 v2.22 and the flags -ax asm5 –secondary = no. Racon-polished consensus sequences were clustered again at 98% identity using the vsearch –cluster_size command and sequences with <10 supporting reads were removed. Another round of polishing was performed using Medaka v1.4.3 (medaka_consensus –m r103_sup_g507). Raw FASTQ files were submitted to NCBI’s sequence read archive under project PRJNA856306 and accession numbers SRR29543362 (d31), SRR29543364 (d43), and SRR29543365 (d45).

For comparison purposes, full-length sequences and partial sequences obtained with MinION and MiSeq, respectively, were aligned using ClustalW in MegAlign 15 (DNASTAR Lasergene 15, Madison, WI, USA), and pairwise distances between consensus sequences were calculated. The full-length *SSU* rRNA gene nucleotide sequences generated in this study were deposited in GenBank under the accession numbers ON932569–ON932571.

### 2.5. Phylogenetic and Pairwise Distance Analysis

The full-length *SSU* rRNA gene nucleotide sequences obtained in this study and appropriate full-length *Blastocystis* reference nucleotide sequences that include all currently accepted subtypes were aligned to generate a phylogenetic tree, which was rooted using *Proteromonas lacertae*, a Stramenopile that is closely related to *Blastocystis*, as an outgroup. Nucleotide sequences were aligned with the Clustal W algorithm, and the phylogenetic analysis was performed using the neighbor-joining (NJ) method, and genetic distances were calculated with the Kimura 2-parameter model using MEGA X [31,32]. A total of 1958 positions were included in the final dataset, which included 74 nucleotide sequences. Bootstrapping with 1000 replicates was used to determine support for the clades generated. Additionally, evolutionary analysis was conducted to establish divergence between nucleotide sequences (pairwise distance) using the Kimura 2-parameter model in MEGA X.

### 2.6. Statistical Analysis

The prevalence of *Blastocystis* was compared between localities, sexes, and age groups via either Fisher’s exact test or chi-squared test using GraphPad Software 9.4.1 (San Diego, CA, USA). Differences were considered significant when *p* < 0.05.

## 3. Results

### 3.1. Prevalence of Blastocystis in Horses

Of the 185 horse fecal samples examined in this study, 81 (43.8%) were found to be *Blastocystis*-positive using PCR and confirmed by NGS (Table 2; Supplementary Table S1). *Blastocystis* was found in all four geographic regions where horses were tested, with prevalence ranging from 41.1% in Costa Atlántica to 56.0% in Bogotá D.C. No difference in overall prevalence was observed among the four localities (*p* = 0.6203).

No difference in overall prevalence was observed between male (42.6%; 29/68) and female horses (44.4%; 52/117) (*p* = 0.8783) or between horses <1 year of age (40.6%; 28/70) and >1 year of age (46.1%; 53/115) (*p* = 0.4478).

### 3.2. Subtypes of Blastocystis in Horses

Twelve STs of *Blastocystis* were detected, ten previously reported (ST1, ST3, ST4, ST5, ST6, ST10, ST14, ST24, ST25, and ST26), and two proposed novel STs, named ST33 and ST34 (Table 2). ST33 was identified in two horses from Llanos Orientales, and ST34 was found in three horses, one from Sabana de Bogotá, and two from Llanos Orientales.

A wide genetic diversity was observed in horses from all regions included in the study, with the lowest diversity of STs in Bogotá D.C. with six STs, and the highest in Llanos Orientales with nine STs detected in 14 and 19 *Blastocystis*-positive horses, respectively (Table 2). Similar genetic diversity was found in females (11 STs) and males (10 STs) (Table 4) as well as in horses younger than 1 year of age (11 STs) and older than 1 year of age (9 STs) (Table 3). ST3, ST4, and ST24 were only present in horses younger than 1 year of age, while ST6 was only found in animals older than 1 year of age. ST6 and ST14 were detected in females but not in males, while ST24 was present in males but not in females.

Mixed ST infections were frequently observed (Table 5; Supplementary Table S1). A single ST was identified in 36 (44.4%) *Blastocystis*-positive horses, while two or more STs were detected in 45 (55.6%) of the *Blastocystis*-positive horses. Multiple combinations of STs were observed, including combinations of two STs and up to six STs observed in three horses from Llanos Orientales. Of these mixed subtype combinations, co-infection with ST10/ST25 (with and without presence of additional STs) was the most observed combination and represented 37.8% (17/45) of all mixed-subtype infections. 

Of the 12 STs identified in the horses, 7 have been previously reported in humans and are potentially zoonotic STs (ST1, ST3, ST4, ST5, ST6, ST10, and ST14). Most *Blastocystis*-positive horses harbored one of those STs (88.9%; 72/81). ST1, ST3, ST4, ST5, ST6, ST10, and ST14 were found in 20, 5, 6, 9, 1, 60, and 3 horses, respectively, as single or mixed STs infections. By location, ST4 (1 horse), ST10 (21 horses), and ST14 (1 horse) were identified in horses from Sabana de Bogotá; ST1 (9 horses), ST5 (1 horse), ST6 (1 horse), ST10 (16 horses), and ST14 (2 horses) in horses from Costa Atlántica; ST1 (1 horse), ST4 (2 horses), ST5 (1 horse), and ST10 (12 horses) in horses from Bogotá D.C.; and ST1 (10 horses), ST3 (5 horses), ST4 (3 horses), ST5 (7 horses), and ST10 (11 horses) in horses from Llanos Orientales. In most *Blastocystis*-positive horses, potentially zoonotic STs were observed as mixed-STs infection (65.4%; 53/81). Only ST1 and ST10 were observed in single ST infections in 28 horses (34.6%; 28/81), ST1 in 3 horses from Costa Atlántica, and ST10 in 11, 7, 5, and 2 horses from Sabana de Bogotá, Costa Atlántica, Bogotá D.C, and Llanos Orientales, respectively.

### 3.3. Blastocystis Intra-Subtype Variation

Fifty-eight unique *Blastocystis* sequences were identified in the 81 *Blastocystis*-positive horses (Table 6). A high intra-subtype variation was observed for ST1 (16 unique sequences among the 50 ST1-positive horses), ST10 (10 unique sequences among the 67 ST10-positive horses), ST26 (9 unique sequences among the 16 ST26-positive horses), ST5 (6 unique sequences among the 9 ST5-positive horses), and ST25 (6 unique sequences among the 29 ST25-positive horses). Fewer sequence variants were observed for the rest of the subtypes, with three unique sequences for ST3, two unique sequences for ST4 and ST14, and one unique sequence for ST6, ST24, ST33, and ST34.

### 3.4. Validation of Novel Subtypes ST33 and ST34

Two sequences generated by Illumina sequencing were suspected of being novel subtypes when compared to nucleotide sequences available in the GenBank database. The sequence similarity between the sequences of the novel subtypes and any nucleotide sequence available in the GenBank database with a subtype designation was approximately 84%. BLAST analysis also identified a sequence from tortoises in the Czech Republic (EF209017) as having approximately 85% sequence similarity with ST33 and ST34. Although both novel subtypes clustered together, their similarity was only 82%.

To confirm validity of the novel subtypes, a nanopore sequencing strategy was used in three horse samples. Samples #d31 and #d43 were used to obtain the full-length sequence of ST33, and sample #d45 was used for ST34. Full-length sequences were successfully obtained for both novel subtypes. There was 100% agreement between the Illumina sequence and the same region within the MinION sequence for both ST33 and ST34.

**Figure 1:**
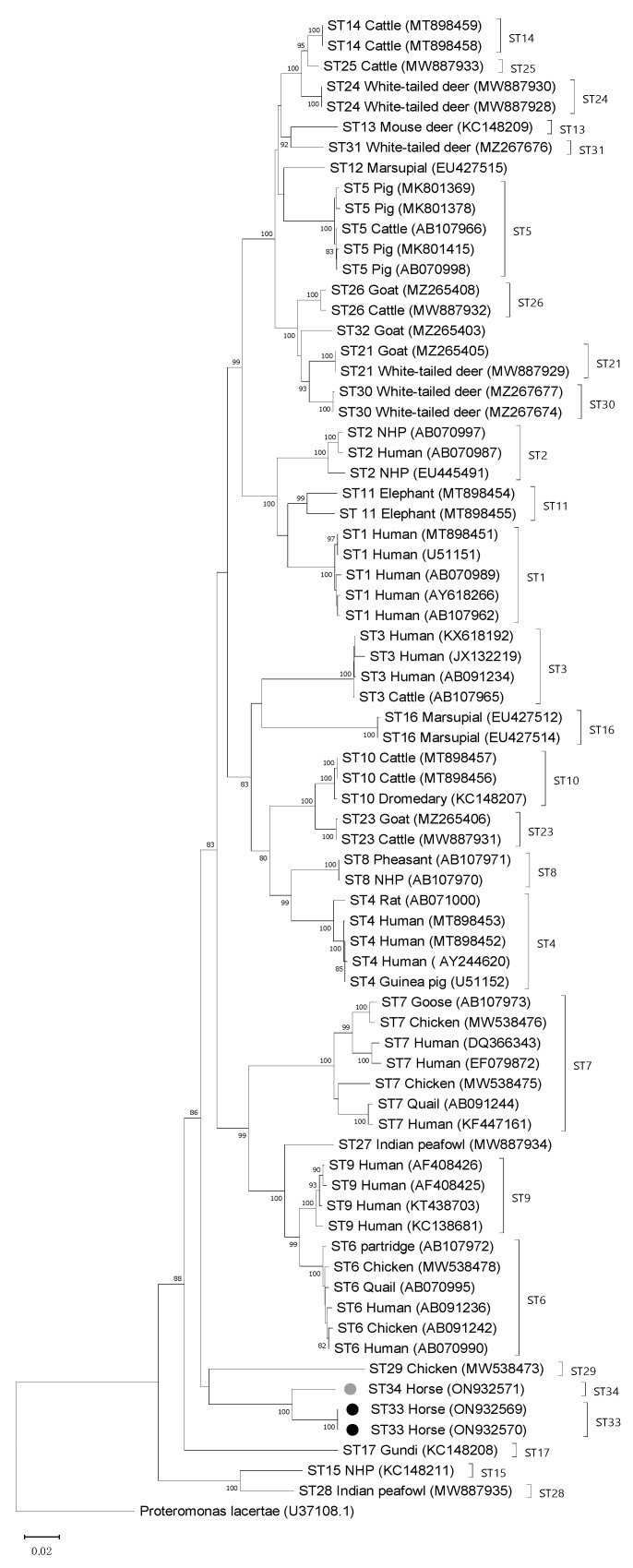
Phylogenetic relationships among *Blastocystis* full-length sequences generated in the present study (novel subtypes are new subtypes ST33 and ST34, which are denoted with black- and gray-filled circles, respectively) and representative reference sequences of all accepted subtypes. *Proteromonas lacertae* was used as outgroup taxon to root the tree. Analysis was conducted by a neighbor-joining method. Genetic distances were calculated using the Kimura two-parameter model. This analysis involved 74 nucleotide sequences, and there were a total of 1953 positions in the final dataset. Bootstrap values lower than 75% are not displayed.

Phylogenetic analysis of full-length sequences using the NJ method showed both ST33 and ST34 clustering with ST29, with a low bootstrap support of 57 (Figure 1). To evaluate the percentage of sequence similarity between ST33 and ST34 and known subtypes, pairwise distance comparisons were used. An 86% similarity was observed for ST33 and ST5 and for ST34 and ST25. The percentage of similarity for both novel STs was 85% or lower when compared with the rest of the known STs (including ST29 at 84%). When full-length sequences of ST33 and ST34 were compared with each other, sequence similarity was 95%.

## 4. Discussion

Horses live in close proximity to humans and are commonly used for companion and agricultural purposes. Yet, data on the subtypes and prevalence of *Blastocystis* in horses is sparse [5]. In this study, 185 samples were screened for *Blastocystis* by PCR, and subtyping was performed using an NGS strategy that can distinguish mixed-subtype infections within individual samples. Using this strategy, we have conducted the largest study of *Blastocystis* ever performed in horses, providing much-needed data to understand prevalence and subtype diversity in this understudied host.

An overall *Blastocystis* prevalence of 43.8% was observed among the 185 horses included in this study, and positive horses were observed in all four collection sites. The high prevalence of *Blastocystis* observed among horses is within the range of other reports from large domestic mammals such as cattle, sheep, goats, and pigs [5]. Presence of *Blastocystis* in horses has only been described in two studies that reported prevalences of 12.5% (1/8) and 54.5% (6/11) in Thailand and Colombia, respectively [7,18]. Five other studies screened horses for *Blastocystis* in Bangladesh, China, Italy, Iran, and Turkey but did not detect it in any of the samples examined [19,20,21,22,23]. Differences in prevalence among studies could be related to many factors, such as those related to the host (breed, age, gender) or diagnostic method used to detect *Blastocystis*. In the study conducted in Thailand, the presence of *Blastocystis* was determine by in vitro cultivation, while in Colombia, PCR was used to detect *Blastocystis* positives. It is noteworthy that out of the six *Blastocystis*-positive horses detected by PCR (6/11; 54.5%), in the Colombian study only two were confirmed as positive using NGS analysis (2/11; 18.2%), thus, 54.5% may be an overestimation of the prevalence in horses in that study [7]. Although more studies from horses are needed to fully describe the prevalence of *Blastocystis* in this host, observations from the present study support *Blastocystis* being common in horses in Colombia. Data from other regions of the world will assist in determining if regional differences in *Blastocystis* prevalence for horses exist.

Horses were host to 12 different STs in this study, and mixed infections were common and observed in 55.6% of positive samples (Table 2 and Table 5). Subtypes observed included ST1, ST3, ST4, ST5, ST6, ST10, ST14, ST24, ST25, ST26, and two proposed novel STs, named ST33 and ST34. Such diversity is similar to what has been reported in livestock such as cattle, goats, and sheep [7,33,34]. However, cattle are ruminants, while horses are monogastric, so the presence of such comparable diversity is quite intriguing. Pigs, another large monogastric domestic mammal, are most frequently host to ST1, ST3, and ST5, although ST2, ST4, ST7, ST8, ST10, and ST15 have also been reported in pigs, albeit with less frequency [35,36,37,38,39,40]. The overlap of subtypes between horses and ruminants could support factors such as an herbivorous diet having as important a role, as physiology influences the colonization of a host with some subtypes of *Blastocystis*. Given that horses, like domestic ruminants, are grazing livestock, it is also possible that shared environmental exposure plays an important role in colonization by individual *Blastocystis* STs. Data on dietary differences and potential reservoir wildlife species that may facilitate transmission to domestic animals are needed to understand such relationships.

Subtype 10 was by far the most common ST among horses in this study and was observed in 60 of the 81 *Blastocystis*-positive horses (Table 6). Subtypes 25 and 26 were also among the most common STs observed and were found in 29 and 16 horses, respectively. Subtypes 10, 25, and 26 are all common STs reported among domestic and wild ruminant hosts [7,9,34,41,42]. Yet, ST14 and ST24, which are also common among ruminants, were quite rare among horses and were observed in only three and one samples, respectively (Table 6). It has been suggested that ST10 and ST14 may be cattle-adapted STs because of their high prevalence among cattle [43]. However, the distribution of these STs among horses may suggest that more than just parasite genetics influence host suitability for a given ST. While more data are needed to confirm associations between horses and any STs, the present study suggests that other factors should be considered in future studies of host specificity of *Blastocystis* STs.

Intra-subtype variability differed between the subtypes identified in horses in this study (Table 6). The most OTUs were observed in ST1 and ST10, with 16 and 10 unique sequences identified in infected horses, respectively. No intra-subtype variation was observed for subtypes ST6, ST24, ST33, and ST34. However, these subtypes were only identified in a few animals, exactly one, one, two, and three horse samples for ST6, ST24, ST33, and ST34, respectively. It is interesting that for ST3, which has usually been found in previous studies to be rather homogeneous [3,44,45], three unique sequences were identified within the five horses infected with ST3. The intra-subtype variability observed in horses in this study may indicate that horses acquire *Blastocystis* from diverse sources in the environment.

Zoonotic infections were common in horses, and 88.9% of *Blastocystis*-positive specimens contained one or more subtypes that have also been reported in humans. Of the potentially zoonotic STs observed in horses in the present study, ST1 was remarkably common and was present in 25% (20/81) of *Blastocystis*-positive horses (Table 6). ST1 was absent from Sabana de Bogotá but was observed in horses from the other three locations (Table 2). It was more common in horses >1 year old, in which it was present in three locations, while it was only observed in Llanos Orientales in <1 year old horses (Table 3). In previous reports of subtype diversity in horses, a single horse from Thailand that was *Blastocystis* positive was reported as having an ST1 infection [18], and in horses from Colombia, ST10, ST14, and ST24 were observed in two horses with ST information [7]. Thus, whether horses are commonly infected with ST1 in other regions of the world remains to be clarified. However, the high rates of ST1 infection among horses in this study indicate they could be an important reservoir of this zoonotic subtype in Colombia. Indeed, ST1 has been reported at high rates among humans in Colombia and is one of the most common STs reported in the Americas [46,47]. Lastly, the high rate of zoonotic ST infection among horses overall indicates more studies on the role of horses in *Blastocystis* transmission to humans are warranted.

Two new STs were observed in this study and following validation through sequencing of the full-length *SSU* rRNA genes and comparison to other named STs, these were given the designations ST33 and ST34. Comparison of the Santin region of the *SSU* rRNA gene for these STs to references available in GenBank did not return any similar sequence matches. However, the barcoding region, which covers approximately the first 600 bp of the gene, did return a BLAST hit with a shared sequence identity of >98% between ST33 and a nucleotide sequence under the GenBank accession number MT039570, which came from a horse in the Czech Republic (unpublished). This same sequence (MT039570) shares 96% sequence identity with ST34 within the same region. The observation of ST33 in a horse from another region of the world further supports the designation of this new ST and may indicate that ST33 and ST34 are part of a horse-specific clade (Figure 1). However, more data from horses and other related hosts would be needed to support such a conclusion.

## 5. Conclusions

This study represents the most comprehensive analysis of *Blastocystis* to ever be conducted in horses. Prevalence and subtype data from this study demonstrate that *Blastocystis* can be common in horses and that many subtypes are able to colonize this host. Our findings have expanded the host range of ST3, ST4, ST5, ST6, ST25, and ST26, as this study represents the first report of these STs in horses. We have identified and validated two new subtypes, ST33 and ST34. Furthermore, horses had subtypes that are frequently observed in both humans and other domestic animals, indicating horses may be reservoirs of these subtypes for other hosts. Given the scarcity of data for *Blastocystis* in horses, this study is an important first step in clarifying the role of horses in the epidemiology of *Blastocystis* and provides essential data for understanding the transmission of different STs between domestic animals and humans in Colombia and beyond.

## Figures and Tables

**Table 1:** Number of horses examined for *Blastocystis* by location with information on age and gender.

Location	No. of Horses	Age		Gender	
		<1 Year	>1 Year	Male	Female
Sabana de Bogotá	59	27	32	26	33
Costa Atlántica	56	10	46	18	38
Bogotá D.C.	25	1	24	1	24
Llanos Orientales	45	32	13	23	22
Total	185	70	115	68	117

**Table 2:** Number of horses examined, number of horses positive, prevalence (%), and subtypes of *Blastocystis* by location. Subtypes in bold denote those previously identified in humans.

Location	No. of Horses Examined	No. of Positives (%)	*Blastocystis* Subtypes: Combinations Observed (No. of Samples)
Sabana de Bogotá	59	25 (42.4%)	**ST4**, **ST10**, **ST14**, ST24, ST25, ST26, ST34: **ST10** (11), ST24(1), ST25 (1), ST26 (1), **ST10**/ST25 (6), **ST10**/ST26 (2), ST25/ST26 (1), **ST10**/ST34(1), **ST4**/**ST10**/**ST14**/ST25/ST26 (1)
Costa Atlántica	56	23 (41.1%)	**ST1**, **ST5**, **ST6**, **ST10**, **ST14**, ST25, ST26: **ST1**(3), **ST10**(7), ST25(2), **ST1**/**ST6**(1), **ST1**/**ST10**(4), **ST1**/ST25(1), **ST10**/**ST14**(2), **ST10**/ST25(1), **ST10**/ST26(1), **ST5**/**ST10**/ST25(1)
Bogotá D.C.	25	14 (56.0%)	**ST1**, **ST4**, **ST5**, **ST10**, ST25, ST26: **ST10**(5), ST25(1), **ST1**/ST25(1), **ST4**/**ST10**(2), **ST5**/**ST10**(1), **ST10**/ST25(1), **ST10**/ST26(2), **ST10**/ST25/ST26(1)
Llanos Orientales	45	19 (42.2%)	**ST1**, **ST3**, **ST4**, **ST5**, **ST10**, ST25, ST26, ST33, ST34: **ST10**(2), ST26(1), ST33(1), **ST1**/**ST5**(1), **ST1**/ST26(1), **ST5**/**ST10**(1), **ST10**/ST25(1), **ST1**/**ST3**/**ST5**(1), **ST1**/**ST3**/**ST10**(1), **ST1**/**ST3**/ST25(1), **ST3**/**ST10**/ST25(1), **ST4**/**ST5**/ST25(1), **ST10**/ST25/ST26(1), **ST1**/**ST5**/ST25/ST26(1), **ST1**/**ST10**/ST26/ST33/ST34(1), **ST1**/**ST3**/**ST4**/**ST5**/**ST10**/ST25(1), **ST1**/**ST4**/**ST5**/**ST10**/ST25/ST26(1), **ST1**/**ST5**/**ST10**/ST25/ST26/ST34(1)
Total	185	81 (43.8%)	

*Blastocystis*-positive samples were observed in all age groups and in both males and females (Table 3 and Table 4 and Supplementary Table S1).

**Table 3:** Number of horses examined, number of horses positive, prevalence (%), and subtypes of *Blastocystis* by age at each location. Subtypes in bold denote those previously identified in humans.

Location	<1 Year		>1 Year	
	No. of Horses Examined/Positive (%)	Subtypes	No. of Horses Examined/Positive (%)	Subtypes
Sabana de Bogotá	27/9 (33.3%)	**ST4**, **ST10**, **ST14**, ST24, ST25, ST26, ST34	32/16 (50.0%)	**ST10**, ST25, ST26
Costa Atlántica	10/2 (20.0%)	**ST10**, **ST14**	46/21 (45.7%)	**ST1**, **ST5**, **ST6**, **ST10**, **ST14**, ST25, ST26
Bogotá D.C.	1/1 (100%)	**ST4**, **ST10**	24/13 (54.0%)	**ST1**, **ST5**, **ST10,** ST25, ST26
Llanos Orientales	32/16 (50.0%)	**ST1**, **ST3**, **ST4**, **ST5**, **ST10**, ST25, ST26, ST33, ST34	13/3 (23.1%)	**ST1**, **ST5**, ST10, ST25, ST26, ST33, ST34
Total	70/28 (40.0%)	**ST1**, **ST3**, **ST4**, **ST5**, **ST10**, **ST14**, ST24, ST25, ST26, ST33, ST34	115/53 (46.1%)	**ST1**, **ST5**, **ST6**, **ST10**, **ST14**, ST25, ST26, ST33, ST34

**Table 4:** Number of horses examined, number of horses positive, prevalence (%), and subtypes of *Blastocystis* by gender at each location. Subtypes in bold denote those previously identified in humans.

	Male		Female	
Location	No. of Horses Examined/Positive (%)	Subtypes	No. of Horses Examined/Positive (%)	Subtypes
Sabana de Bogotá	26/12 (46.2%)	**ST10**, ST24, ST25, ST26, ST34	33/13 (39.4%)	**ST4**, **ST10**, **ST14**, ST25, ST26,
Costa Atlántica	18/6 (33.3%)	**ST1**, **ST5**, ST10, ST25	38/17 (44.7%)	**ST1**, **ST6**, **ST10**, **ST14**, ST25, ST26
Bogotá D.C.	1/0 (0%)		24/14 (58%)	**ST1**, **ST4**, **ST5**, **ST10**, ST25, ST26
Llanos Orientales	23/11 (47.8%)	**ST1**, **ST3**, **ST4**, **ST5**, **ST10**, ST25, ST26, ST33	22/8 (36.4%)	**ST1**, **ST3**, **ST5**, **ST10**, ST25, ST26, ST33, ST34
Total	68/29 (42.6)	**ST1**, **ST3**, **ST4**, **ST5**, **ST10**, ST24, ST25, ST26, ST33, ST34	117/52 (44.4%)	**ST1**, **ST3**, **ST4**, **ST5**, **ST6**, **ST10**, **ST14**, ST25, ST26, ST33, ST34

**Table 5:** Combination of STs observed in horses in Colombia at each location. Subtypes in bold denote those previously identified in humans.

Location	Subtypes (Number of Samples)					
	1 Subtype	2 Subtypes	3 Subtypes	4 Subtypes	5 Subtypes	6 Subtypes
Sabana de Bogotá	**ST10**(11) ST24(1) ST25(1)ST26(1)	**ST10**/ST25(6) **ST10**/ST26(2) ST25/ST26(1) **ST10**/ST34(1)			**ST4**/**ST10**/**ST14**/ST25/ST26 (1)	
Costa Atlántica	**ST1**(3) **ST10**(7) ST25(2)	**ST1**/**ST6**(1) **ST1**/**ST10**(4) **ST1**/ST25(1) **ST10**/**ST14**(2) **ST10**/ST25(1) **ST10**/ST26(1)	**ST5**/**ST10**/ST25(1)			
Bogotá D.C.	**ST10**(5) ST25(1)	**ST1**/ST25(1) **ST4**/**ST10**(2) **ST5**/**ST10**(1) **ST10**/ST25(1) **ST10**/ST26(2)	**ST10**/ST25/ST26(1)			
Llanos Orientales	**ST10**(2) ST26(1) ST33(1)	**ST1**/**ST5**(1) **ST1**/ST26(1) **ST5**/**ST10**(1) **ST10**/ST25(1)	**ST1**/**ST3**/**ST5**(1) **ST1**/**ST3**/**ST10**(1) **ST1**/**ST3**/ST25(1) **ST3**/**ST10**/ST25(1) **ST4**/**ST5**/ST25(1) **ST10**/ST25/ST26(1)	**ST1**/**ST5**/ST25/ST26(1)	**ST1**/**ST10**/ST26/ST33/ST34 (1)	**ST1**/**ST3**/**ST4**/**ST5**/**ST10**/ST25(1) **ST1**/**ST4**/**ST5**/**ST10**/ST25/ST26(1) **ST1**/**ST5**/**ST10**/ST25/ST26/ST34(1)
Total	**ST1**(3) **ST10**(25) ST24(1) ST25(4) ST26(2) ST33(1)	**ST1**/**ST5**(1) **ST1**/**ST6**(1) **ST1**/**ST10**(4) **ST1**/ST25(2) **ST1**/ST26(1) **ST4**/**ST10**(2) **ST5**/**ST10**(2) **ST10**/**ST14**(2) **ST10**/ST25(9) **ST10**/ST26(5) ST25/ST26(1) **ST10**/ST34(1)	**ST1**/**ST3**/**ST5**(1) **ST1**/**ST3**/**ST10**(1) **ST1**/**ST3**/ST25(1) **ST3**/**ST10**/ST25(1) **ST4**/**ST5**/ST25(1) **ST5**/**ST10**/ST25(1) **ST10**/ST25/ST26(2)	**ST1**/**ST5**/ST25/ST26(1)	**ST1**/**ST10**/ST26/ST33/ST34(1) **ST4**/**ST10**/**ST14**/ST25/ST26(1)	**ST1**/**ST3**/**ST4**/**ST5**/**ST10**/ST25(1) **ST1**/**ST4**/**ST5**/**ST10**/ST25/ST26(1) **ST5**/**ST10**/ST25/ST26/ST34(1)

**Table 6:** *Blastocystis* subtypes identified in horses including number of positive horses in which each subtype was identified and number of unique sequences among each subtype.

*Blastocystis* Subtype	No. of *Blastocystis*-Positive Horses	No. of Unique Sequences Per Subtype
ST1	20	16
ST3	5	3
ST4	6	3
ST5	9	6
ST6	1	1
ST10	60	10
ST14	3	2
ST24	1	1
ST25	29	6
ST26	16	7
ST33	2	1
ST34	3	1

## Data Availability

All relevant data are within the article and its additional files. The raw FASTQ files were submitted to NCBI’s sequence read archive under project PRJNA856306. The sequences data were submitted to the GenBank database under the accession numbers ON932503–ON932559 (NGS) and ON932569–ON932571 (MinION).

## Supplementary Materials

The following supporting information can be downloaded at: https://www.mdpi.com/article/10.3390/microorganisms10091693/s1.
